# Exploration of factors mediating the effect of socioeconomic deprivation on rates of childhood stunting in Sefton: an ecological analysis

**DOI:** 10.1093/pubmed/fdag047

**Published:** 2026-07-06

**Authors:** Timothy Martin, Anna Head, Philip McHale

**Affiliations:** University of Liverpool, Department of Public Health, Policy and Systems, Waterhouse Building, Liverpool, L69 3GB, United Kingdom; Sefton Council, Public Health Team, Magdalen House, 30 Trinity Road, Bootle, Sefton, L20 3NJ, United Kingdom; University of Liverpool, Department of Public Health, Policy and Systems, Waterhouse Building, Liverpool, L69 3GB, United Kingdom; University of Liverpool, Department of Public Health, Policy and Systems, Waterhouse Building, Liverpool, L69 3GB, United Kingdom

**Keywords:** children, epidemiology, socioeconomics factors

## Abstract

**Background:**

Childhood height is an important indicator of child health. Stunting is associated with poor health outcomes, with consistent socioeconomic inequalities seen. This study aims to generate hypotheses for causal mechanisms through which socioeconomic deprivation leads to stunting.

**Methods:**

A multiyear cross-sectional study using National Childhood Measurement Programme data from 2013/2014 to 2022/2023 in the Metropolitan Borough of Sefton, and ecological exposures for hypothesised mediators. We used logistic regression models to estimate the effect of socioeconomic deprivation on stunting rates and generate hypotheses for causal pathways.

**Results:**

There was a significantly higher odds ratio for stunting in the most deprived quintile compared to the least deprived in children aged 4–5 years (1.60; 95% CI 1.10–2.35) and 10–11 years (2.02; 95% CI 1.28–3.25) after adjustment for confounders. In children aged 10–11 years 75.3% of this effect was attenuated when hypothesised mediators low birth weight, preterm birth, breastfeeding rates, food insecurity and healthcare access, were included; this result was not statistically significant. No attenuation was demonstrated in children aged 4–5 years.

**Conclusions:**

This research shows an association between socioeconomic deprivation and higher stunting rates in Sefton, which was non-significantly attenuated after adjustment for hypothesised mediators in children aged 10–11 years.

## Background

Childhood growth is an important indicator of a child’s health.^1^ Stunting is a measure of poor growth, defined as height-for-age greater than two standard deviations below the population median according to WHO growth reference standards.^2^^,^^3^ Previous research has shown that stunting is associated with recurrent childhood infections, worse neurodevelopmental and educational outcomes,^4^ and increased risk of chronic health problems and premature mortality.^5^ Addressing the causes of stunting may help prevent these negative effects in later life.^6^ Studies in the UK have shown significant associations between socioeconomic deprivation and lower childhood height.^1^^,^^7–9^ From the mid-20th to the early 21st century, childhood height in the UK increased for all socioeconomic strata, but most for those living in the highest levels of socioeconomic deprivation.^1^^,^^7^^,^^8^

The trend of increasing childhood height in the UK has started reversing, with the average height of children aged 5 decreasing between 2013 and 2019.^10^ The Food Foundation suggests that austerity policies driving changes in broader socioeconomic conditions, such as reduced affordability of healthy diets, could be causing this decline in childhood heights.^11^ It is in this context that it is important to understand the causal pathways between socioeconomic deprivation and childhood stunting. A mediator is an intermediate on the causal pathway between an exposure and an outcome.^12^ Mediators must be at least in part caused by the exposure and, in turn, have a causal effect on the outcome. Existing evidence on causal pathways using individual-level data suggests low breastfeeding rates,^13^^,^^14^ low birth weight,^15–18^ preterm birth,^19^^,^^20^ food insecurity,^21^^,^^22^ and lack of access to healthcare^13^^,^^23^^,^^24^ as potential mediators for the effect of socioeconomic deprivation on childhood stunting.

This study aims to examine the relationship between socioeconomic deprivation and childhood stunting in the Metropolitan Borough of Sefton, and to generate hypotheses about causal mechanisms for the effect of socioeconomic deprivation on childhood stunting in the local area.

## Methods

### Study design

A multi-year cross-sectional study using National Childhood Measurement Programme (NCMP) data and ecological exposures with an exploratory mediation-style analysis.

### Study population

The study used anonymized data from children in reception (4–5 years) and year 6 (10–11 years) attending state schools and living in the Sefton area who participated in the NCMP between 2013/14 and 2022/23. The NCMP measures the height and weight of all children attending state primary schools in England at reception and year 6.^25^ NCMP data provides information on each pupil’s Lower Super Output Area (LSOA) (an administrative area with an average population of 1500), Middle Super Output Area (MSOA) (an administrative area consisting of four or five LSOAs) and electoral ward (an electoral division with an average population of 5500), allowing for linkage to other data sources available at a small area level. Sefton is a Metropolitan Borough in the northwest of England, which has a mixture of deprived urban and affluent suburban and rural areas.

### Outcome

The NCMP dataset provides height-for-age z scores for all children with height measurements, from which children with stunted growth can be identified. Stunting is classified as height-for-age greater than two standard deviations below the population median defined by the WHO growth reference standard.^3^

### Exposure

Exposure to socioeconomic deprivation was measured by 2019 Indices of Deprivation Affecting Childhood (IDACI). The 2019 IDACI is an ecological measure of the proportion of children aged 0 to 15 years living in families with income deprivation available at LSOA level.^26^ LSOAs in Sefton were ranked according to IDACI score and divided into quintiles.

### Directed acyclic graph

**Figure 1:**
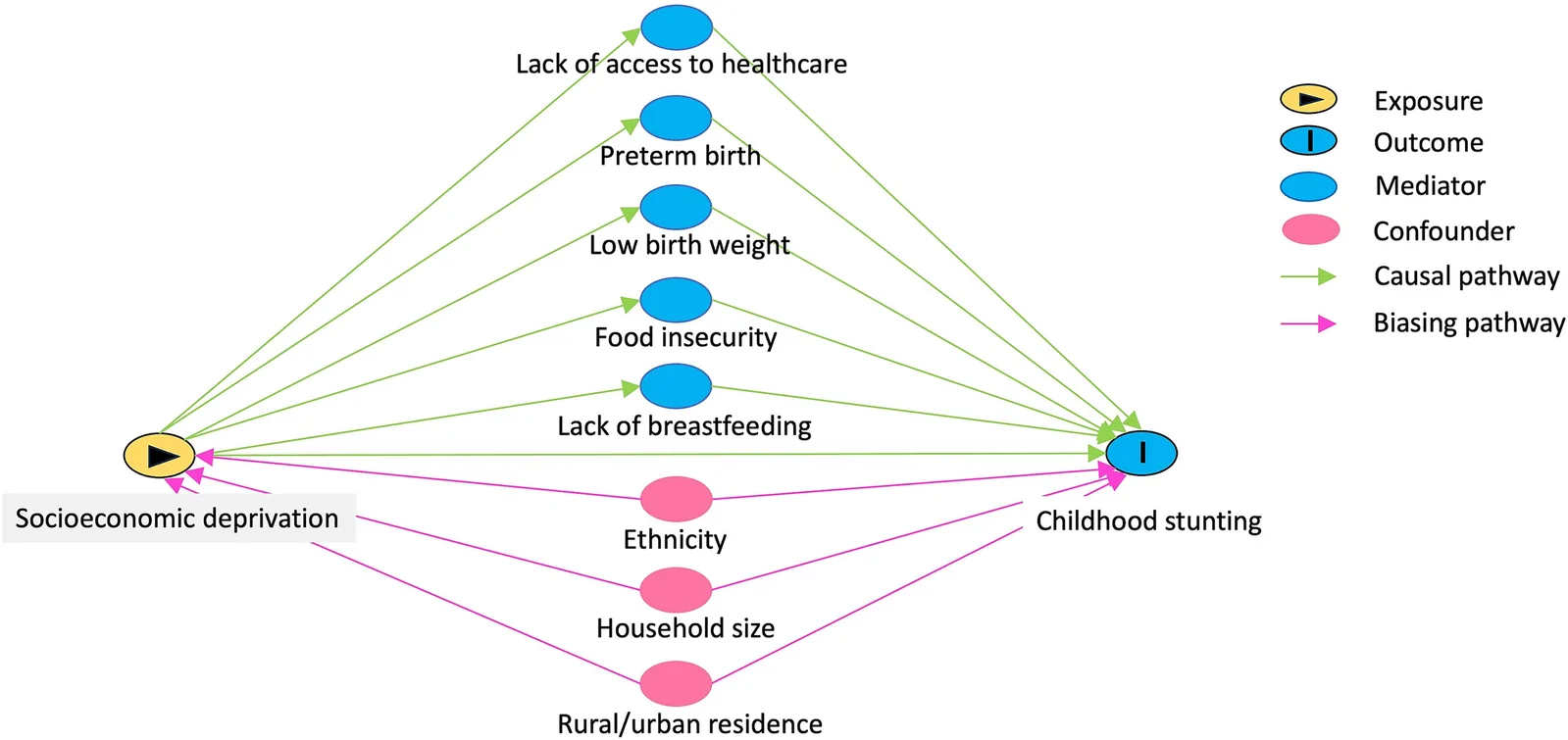
DAG for causal pathways between socioeconomic status and childhood stunting as identified by the literature review.

We conducted a literature review (see Appendix 1) to inform the production of a Directed Acyclic Graph (DAG) to establish potential causal pathways between socioeconomic deprivation and childhood stunting and ascertain possible confounding factors (Fig. 1). Informed by this DAG, hypothesised mediators low birth weight, preterm birth, lack of breastfeeding, food insecurity and lack of access to healthcare were included in an exploratory mediation-style analysis. Potential confounding factors included were ethnicity,^1^^,^^7^ urban/rural residence,^22^^,^^23^ and household size.^19^

### Hypothesised mediators

Ecological data sources for mediator and confounder variables are shown in Appendix 2. Low birth weight was defined as below 2500 grams. Preterm birth was defined as birth before 37 weeks gestation. Information on low birth weight and preterm births was available at MSOA level. Breastfeeding rates at six to eight weeks after birth were available at electoral ward level. Food insecurity was measured by the Priority Places for Food Index version 2 (PPFIv2) score 2024, which was available at LSOA level.^27^ PPFIv2 is an ecological multicomponent measure of food insecurity, which includes availability of retail food stores and socioeconomic barriers. Access to healthcare was measured by the health domain score of the index of Access to Healthy Assets & Hazards version 2 (AHAHv2) 2017 which was available at LSOA level.^28^ The health domain of AHAHv2 is a multicomponent ecological measure of local level access to health facilities.

### Confounding factors

Confounding factors included were ethnicity, rural/urban residence and household size. Ethnicity data was available as a percentage of the population at LSOA level, this was divided in to six main groups: Asian, Black, Mixed, White British, White Other, and Other. Data on urban/rural residence were available for each pupil at LSOA level. There was no small area-level information on household sizes in Sefton. Instead, data on household overcrowding was used, which was available at LSOA level.

### Statistical analysis

#### Descriptive statistical analysis

Chi-squared test for trend was used to test for associations between categorical variables and IDACI quintiles where assumptions were met. A Fisher’s Exact Test was used for categorical variables that did not meet the criteria for the Chi-squared test. An Analysis of Variance (ANOVA) test was used to test for statistically significant differences between the IDACI quintiles for continuous variables. A Mann-Kendall test was performed to test for statistically significant trends in rates of stunting over time.

The reception (aged 4–5) and year 6 groups (aged 10–11) were analysed separately as there were significant differences between the IDACI quintiles according to year groups. Unadjusted estimates for the Slope Index of Inequality (SII) and Relative Index of Inequality (RII) were calculated for reception and year 6 groups to show the social gradient of childhood stunting according to IDACI quintiles. A logistic regression model was used to examine the association with deprivation according to IDACI quintiles and the rates of childhood stunting after adjustment for confounding factors in both the reception and year 6 groups.

#### Exploratory mediation-style analysis

An exploratory mediation-style analysis was conducted using the proportion eliminated approach. The proportion eliminated after adjustment for ecological indicators consistent with hypothesised pathways was calculated using the total effects Odds Ratio (OR) and the direct effects OR shown in the formula below. The total effects OR was calculated using a logistic regression model showing the association between deprivation according to IDACI and stunting after adjustment for confounders (ethnicity, rural/urban residence and household overcrowding), but before hypothesised mediators were included. The logistic regression model to calculate the direct effects OR for the effect of deprivation on stunting included hypothesised mediators (low birth weight, preterm birth, lack of breastfeeding, food insecurity, and lack of access to healthcare) in addition to socioeconomic deprivation and confounders.


\begin{align*} \mathrm{Proportion}\ \mathrm{Eliminated}=&\,\left({\mathrm{OR}}_{\mathrm{total}\ \mathrm{effects}}\hbox{--} {\mathrm{OR}}_{\mathrm{direct}\ \mathrm{effects}}\right)/\\&\left({\mathrm{OR}}_{\mathrm{total}\ \mathrm{effects}}\hbox{--} 1\right) \end{align*}

### Missing data

**Figure 2:**
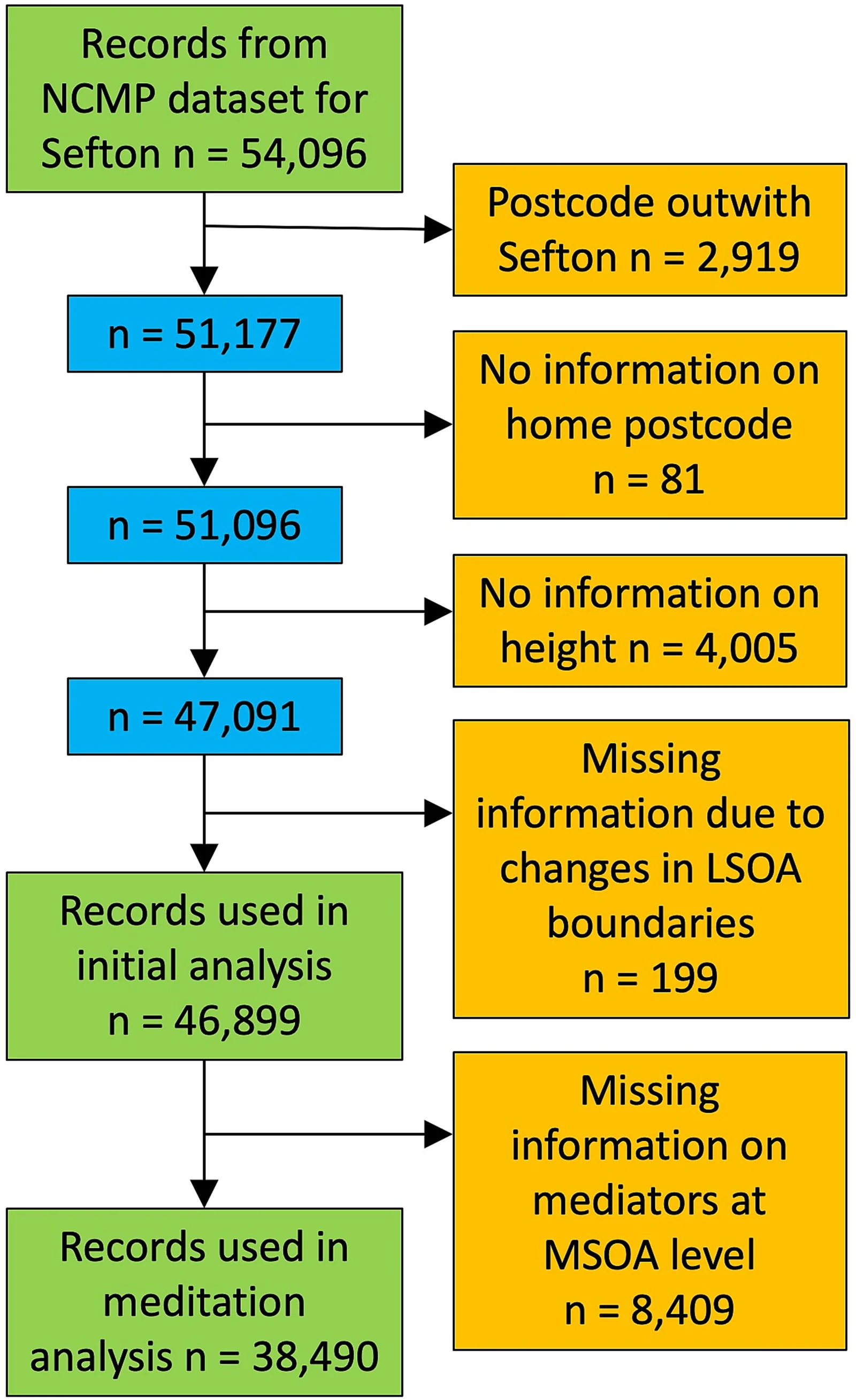
Flowchart of the reasons for exclusion from and number of records used in the analysis.

Figure 2 shows participants in the Sefton NCMP dataset who were included and excluded from the analysis. Participants who lived out with Sefton were excluded, as were those with missing data for stunting status or LSOA. Data for mediators’ low birth weight and preterm birth were not available for some MSOAs, as they were suppressed due to small numbers for confidentiality reasons. Participants in areas without information on hypothesised mediator variables were excluded from the exploratory mediation-style analysis.

## Results

### Sample characteristics

Records for 46 899 children from the NCMP dataset in Sefton from 2013/2014 to 2022/2023 (86.7% of total records available) were included in the initial analyses, adjusted for confounders. A reduced sample of 38 490 cases (71.2%) was included in the exploratory mediation-style analysis due to missing data for some variables. Table 1 Shows differences in characteristics according to IDACI quintiles, including demographics and study variables.

**Table 1:** Demographics, confounders and hypothesised mediators according to IDACI quintile.

	IDACI Quintiles					*P*-value
	1 (most deprived) (*n* = 11 006)	2 (*n* = 10 077)	3 (*n* = 9574)	4 (*n* = 8523)	5 (least deprived) (*n* = 7911)	
**Primary outcome**						
**Stunting**						
Stunting (%)	2.05% (*n* = 226)	1.40% (*n* = 141)	1.07% (*n* = 102)	1.13% (*n* = 96)	1.07% (*n* = 85)	<.0005^*^
**Demographics**						
**Sex**						
Female (%)	49.48% (*n* = 5446)	48.97% (*n* = 4935)	48.32% (*n* = 4626)	48.47% (*n* = 4228)	48.62% (*n* = 3846)	.339^*^
Male (%)	50.52% (*n* = 5560)	51.03% (*n* = 5142)	51.68% (*n* = 4928)	51.53% (*n* = 4295)	51.38% (*n* = 4065)	.339^*^
**School year**						
Reception (%)	51.40% (*n* = 5657)	49.83% (*n* = 5021)	48.66% (*n* = 4658)	46.50% (*n* = 3963)	45.72% (*n* = 4294)	<.0005^*^
Year 6 (%)	48.60% (*n* = 5349)	50.17% (*n* = 5056)	51.34% (*n* = 4916)	53.50% (*n* = 4560)	54.28% (*n* = 3617)	<.0005^*^
**Potential confounders**						
**Ethnicity**						
Asian (%)	1.91%	1.75%	1.33%	1.07%	1.15%	<.0005^**^
Black (%)	0.94%	0.57%	0.48%	0.27%	0.23%	<.0005^**^
Mixed (%)	1.64%	1.66%	1.34%	1.24%	1.31%	<.0005^**^
White British (%)	88.86%	90.22%	93.64%	94.41%	94.77%	<.0005^**^
White Other (%)	5.41%	5.07%	3.13%	2.63%	2.26%	<.0005^**^
Other (%)	1.25%	0.72%	0.47%	0.38%	0.28%	<.0005^**^
**Rural/urban residence**						
Urban (%)	100.00% (*n* = 11 006)	98.59% (*n* = 9935)	99.24% (*n* = 9549)	99.40% (*n* = 8472)	96.93% (*n* = 7668)	<.0005^***^
Rural (%)	0.00% (*n* = 0)	1.41% (*n* = 142)	0.26% (*n* = 142)	0.60% (*n* = 51)	3.07% (*n* = 243)	<.0005^***^
**Household overcrowding**						
Household with overcrowding by room occupancy (%)	7.16%	5.60%	3.82%	2.87%	2.48%	<.0005^**^
**Potential mediators**						
**Low birth weight**						
Percentage of births < 2500 grams (%)	12.12%	10.26%	8.15%	7.69%	8.84%	<.0005^**^
**Preterm birth**						
Percentage of births < 37 weeks gestation (%)	12.28%	10.86%	9.04%	7.82%	9.26%	<.0005^**^
**Breastfeeding**						
Percentage breastfeeding at 6–8 weeks (%)	28.22%	37.00%	39.62%	43.80%	44.16%	<.0005^**^
**Food insecurity**						
PPFIv2 mean score (lower score indicates greater food insecurity)	5725	8971	11 194	14 862	21 500	<.0005^**^
**Access to health service**						
Health domain AHAHv2 mean score (higher score indicates lower access to healthcare)	6.40	9.07	9.41	10.02	13.35	<.0005^**^

^*^ *P*-value calculated using Chi-squared for trend test, ^**^P-values calculated using ANOVA test, ^***^P-values calculated using Fisher’s exact test.

There were no significant changes in the stunting rates from 2013/14 to 2022/23 in either age group (see Appendix 3).

### Inequalities in childhood stunting according to IDACI quintile

In unadjusted absolute numbers, one in 41 reception children from the most deprived IADCI quintile had stunted growth, compared to one in 72 from the least deprived IDACI quintile. In year 6 pupils, one in 62 children in the most deprived IDACI quintile had stunted growth, compared with one in 122 pupils in the least deprived quintile (full results from all IDACI quintiles shown in Appendix 4). The unadjusted SII of stunting prevalence according to IDACI quintile was −1.43 (95% CI –2.04, −0.83) in the reception group, and −0.90 (95% CI –1.40, −0.40). This indicates stunting rates are 1.43 percentage points higher in the most compared to the least deprived IDACI quintile in reception pupils, and among year 6 pupils, 0.9 percentage points higher in the most deprived quintile. The unadjusted RII for stunting were 2.52 (95% CI 1.67, 4.03) for the reception group and 2.36 (95% CI 1.43, 4.17) for the year 6 group. This shows that stunting prevalence in the most deprived IDACI quintile compared to the least is 2.52 times higher in the reception group and 2.36 times higher in year 6 pupils.

Logistic regression models were performed for reception (4–5 years) and year 6 (10–11 years) groups, examining the relationship between deprivation according to IDACI quintiles and rates of stunting after adjusting for confounders (see Appendix 4). In the reception group, the adjusted OR for stunting was 1.60 (95% CI 1.10, 2.35; *P* = .016) among children living in the most deprived IDACI quintile compared to those living in the least deprived quintile. In the year 6 group, the adjusted OR for stunting was 2.02 (95% CI 1.28, 3.25; *P* = .003), higher for the most deprived IDACI quintile than the least.

### Exploratory mediation-style analysis

**Figure 3:**
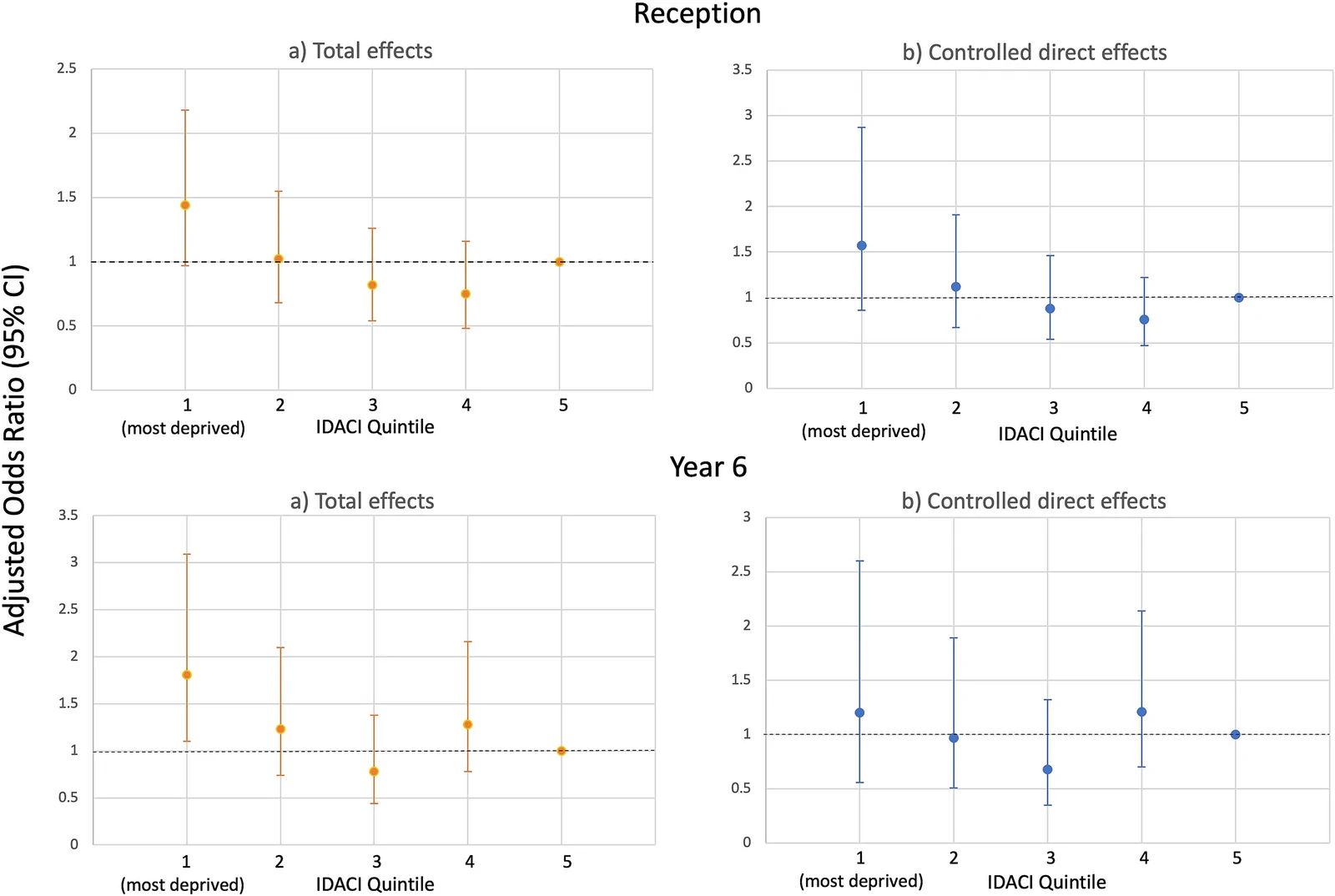
Adjusted odds ratios for the relationship between IDACI quintile and stunting, adjusted for (a) confounders only (total effects model); (b) confounders and mediators (controlled direct effects) in reception and year 6 groups.

The proportion of the effects of deprivation on rates of stunting attenuated through the hypothesised mediators (low birth weight, preterm birth, lack of breastfeeding, food insecurity, and lack of access to healthcare) was calculated using the proportion eliminated approach (see Fig. 3; full exploratory mediation-style analysis results shown in Appendix 5).

In the reception group, the hypothesised mediators did not attenuate any of the effects of deprivation on childhood stunting. In the year 6 group, 75.3% of the effect of deprivation on childhood stunting was attenuated after the inclusion of the ecological indicators consistent with hypothesised mediators. However, in both the reception and year 6 groups, the ORs in both models had a wide 95% CI, which crossed 1, so the results were not statistically significant.

## Discussion

### Main findings of this study

In this cross-sectional study of child measurement data in the northwest of England, we explored the effect of socioeconomic deprivation on stunting rates in a logistic regression model, adjusting for confounders of ethnicity, household overcrowding, and urban/rural residence. For both age groups studied (age 4–5; age 10–11), children living in the most deprived IDACI quintile had a higher odds of stunted growth compared to the least deprived quintile. An exploratory mediation-style analysis was performed to examine whether the effect of socioeconomic deprivation on childhood stunting in Sefton would be attenuated after adjustment for ecological indicators consistent with hypothesised mediators (low birth weight, preterm birth, lack of breastfeeding, food insecurity and lack of access to healthcare). In the reception group (age 4–5), none of the effects of socioeconomic deprivation on childhood stunting was attenuated by the inclusion of hypothesised mediators, whilst in the year 6 group (age 10–11), over three-quarters of the effect of socioeconomic deprivation on childhood stunting was attenuated. However, results were not statistically significant and chance may have accounted for this difference between age-groups as the confidence intervals in both groups were wide.

### What is already known on this topic

Our findings align with research by Orr *et al*.^1^ and Hancock *et al*.^7^ showing a significant correlation between socioeconomic deprivation and higher rates of childhood stunting in the UK. Bann *et al*.^8^ and Silverwood *et al*.^9^ also show a significant association between socioeconomic deprivation and lower childhood height in the UK. Stunting has many long-term health effects, including poorer cognitive and educational outcomes and increased premature mortality.^4^^,^^5^ Reducing socioeconomic inequalities in stunting rates could reduce health inequalities more generally.^29^

Limited research has examined the causal pathways from socioeconomic deprivation to childhood stunting. In Scotland, Silverwood *et al*.^9^ conducted a mediation analysis examining how low birth weight could mediate the effect of socioeconomic deprivation on lower childhood height. Using a traditional mediation analysis, they showed low birth weight mediated around 40% of the effect of reduced childhood height in the most deprived compared to the least deprived quartile according to the Scottish Index for Multiple Deprivation, which was statistically significant. There is a lack of research on other potential mediators for the effect of socioeconomic deprivation on childhood stunting.

### What this study adds

This study provides further evidence of the ongoing inequalities in childhood stunting rates according to socioeconomic deprivation in the UK, using secondary data from the NCMP—a large, representative and reliable source of information on childhood height which includes most children attending state schools in England. Using confounding and potential mediating factors identified by prior literature,^30^ this cross-sectional and ecological research has also shown possible attenuation of this effect through hypothesised mediators low birth weight, preterm birth, low breastfeeding rates, lack of access to healthcare and food insecurity in year 6 pupils. Individual-level longitudinal research is required to strengthen the evidence on the causal pathways through which socioeconomic deprivation causes childhood stunting and highlight areas where interventions could be introduced to tackle childhood stunting and reduce associated health problems.

### Limitations of this study

This was a cross-sectional study, so it cannot show temporality between an exposure and an outcome, and thus should not be used to infer causality.^31^ The cross-sectional data from the NCMP were collected over ten years, during which time the exposures may have changed. Most ecological exposure variables used data from the last five years, so they may not accurately represent exposure for the study period.

This study used small area ecological exposure variables, applying this to each pupil’s data to assess for an association between the exposure and individual pupils’ outcome, stunting or no stunting.^32^ This means we cannot establish whether each individual had a particular exposure, so it is not possible to confirm individual-level association between the exposure and the outcome; this is known as the ecological fallacy.^33^ The results from ecological analyses such as this study should be treated with caution and cannot be used to infer causality. Ecological studies can be used to generate new hypothesis but these should be confirmed using individual-level studies.^31^ Some variables in this study were only available at higher ecological levels, such as MSOA and electoral ward level, increasing the chances of the ecological fallacy.^33^

Access to healthcare was measured at an ecological level by the health domain score of the AHAHv2. From the DAG (see Fig. 1) informed by the literature review, it was expected access to healthcare would be better in areas of lower socioeconomic deprivation. According to the health domain of the AHAHv2, access to healthcare was higher in the most deprived areas in Sefton. This indicator used proximity to health facilities to measure access, which may be better in deprived urban areas which are densely populated than more affluent rural or suburban areas. There will likely be other socioeconomic barriers to access to healthcare for people living in greater socioeconomic deprivation which are not captured by this ecological measure.

Due to suppression of small numbers for confidentiality, there was missing data on preterm and low birth weight for eight of the 39 MSOAs within Sefton. Children from these areas were excluded from the exploratory mediation-style analysis, decreasing the sample for the analysis by 17.9% and reducing its statistical power. In addition, excluding areas with small total numbers of preterm and low birth weight babies may have biased the results of the analysis, as these were the areas with the lowest rates of low birth weight and preterm birth. The areas with suppressed data had significantly lower levels of deprivation, a lower percentage of children with stunting, higher white British ethnicity, higher breastfeeding rates, higher rural residence, higher food insecurity and lower access to healthcare compared with the children included in the exploratory mediation-style analysis (see Appendix 6). Exclusion of these areas is likely have reduced the effect attenuated by the proposed mediators.

A review of the literature (Appendix 1) was conducted to identify potential causal pathways between socioeconomic deprivation and childhood stunting. This literature review was used to inform the creation of a DAG with confounders and potential mediators for this effect. Due to limitations in the available literature, it is possible that potential mediators and confounders could have been misclassified. This could have led to over- or under-adjustment of results, reducing the reliability of the results.

There is evidence from previous studies that household size is a potential confounder of the effect of socioeconomic deprivation on childhood stunting. As this data was not available for Sefton, we used household overcrowding based on room occupancy as a proxy for household size. However, the two measures are not directly comparable, as household overcrowding is related to the size of the accommodation as well as the household size. Household overcrowding may also be associated with socioeconomic deprivation, so it could also be either a mediator or a collider rather than a confounder on the pathways between socioeconomic deprivation and childhood stunting. Adjusting for this could represent an over-adjustment and affect the results of the analysis.

## Conclusions

Our study found a significant association between socioeconomic deprivation and higher rates of stunting in Sefton. In children aged 10–11 years, some of the effects of socioeconomic deprivation on childhood stunting were attenuated after adjustment for ecological indicators consistent with hypothesised pathways. However, caution is needed in interpreting these results, as this is a cross-sectional ecological analysis, so it cannot be used to infer causality. Research using individual-level longitudinal study designs is needed to examine the effect of mediators in explaining the socioeconomic inequalities in childhood stunting and identify interventions for reducing inequalities in childhood stunting and its long-term health impacts.

## Supplementary Material

Supplementary_material_fdag047

## Data Availability

Data supporting the findings of this study can be made available on reasonable request.

## Notes

**Martin Timothy**, Public Health Registrar

**McHale Philip**, Senior Clinical Lecturer

**Head Anna**, Research Fellow
