# Supplementary material for: Exploration of factors mediating the effect of socioeconomic deprivation on rates of childhood stunting in Sefton: an ecological analysis
Source: J Public Health (Oxf). 2026 Jul 6;48(3):788–96. doi: 10.1093/pubmed/fdag047 (PMC13531711; doi:10.1093/pubmed/fdag047)
Supplement: Supplementary file 1 [file supplementary_material_fdag047.zip › Appendix 1 Literature review on the causal pathways between socioeconomic deprivation and childhood stunting from OECD countries.docx]

# Appendix 1: Literature review on the causal pathways between socioeconomic deprivation and childhood stunting from OECD countries

Table 2: PECO framework for literature search

| Population | Children living in OECD countries |
| --- | --- |
| Exposure | High socioeconomic deprivation |
| Comparison | Low socioeconomic deprivation |
| Outcome | Childhood stunting/height |

Table 3: Literature search terms and limits used

| Search order | Concept | Search terms |
| --- | --- | --- |
| Search 1 | Stunting | “Stunt*” or “faltering growth” or “linear growth retard*” or “height for age deficit” or “short stature” |
| Search 2 | Socioeconomic factors | “socio*economic*” or “deprivation” or “inequality” |
| Search 3 | Children | “Infant*” OR “child*” |
| Search 4 | Mediator | "path*" OR "mediat*" OR "associat*" OR "factor*” OR “caus*” |
| Restrictions | Children (0-19 years) only, English language only, journal articles only, publication years 2014-2024 | |

*Figure 4: A flow diagram of the studies identified by the literature search, screened, reviewed and included in the literature review*

Table 4: Summary of studies included in the literature review

| Author, year, title, country | Design,  Participants, sample size | Socioeconomic status (SES), covariates, potential mediators | Outcome measure(s) | Key Findings |
| --- | --- | --- | --- | --- |
| **Akpinar & Teneler. 2023**  Negative birth outcomes and stunting among adolescent and non-adolescent mothers in Türkiye.  Turkey | **Design:**  Cross-sectional study.  **Participants:**  Mothers (15-49 years) child (0-5 years) pairs from 2018 Demographic and Health Survey.  **Sample size:**  2755 mother-child pairs. | **SES:**  Maternal education, welfare status.  **Covariates:**  Maternal age (adolescent vs non-adolescent),  antenatal visits, gender, rural/urban residence, region.  **Potential mediators:**  Low birth weight, preterm. | **Height:**  Stunting as defined by the 2006 WHO growth standard reference. | Multivariate logistic regression for factors associated with stunting showed significant associations with lower welfare status (adjusted OR 2.22 (95% CI 1.47-3.50), p<0.01) and low birth weight (adjusted OR 2.86 (95% CI 1.90-4.30), p<0.01). No significant association was seen with maternal age or education.  Multivariate logistic regression for term low birth weight showed significant association with lower welfare status (adjusted OR 2.09 (95% CI 1.44, 3.02), p<0.01). |
| **Aldana-Parra et al. 2020**  Associations between maternal BMI, breastfeeding practices and infant anthropometric status in Colombia; secondary analysis of ENSIN 2010.  Colombia | **Design:**  Cross-sectional study.  **Participants:**  Term singleton children under two years of age and their mothers. Part of the 2010 National nutritional survey.  **Sample Size:**  5,030 child-mother pairs. | **SES:**  Wealth index, maternal education, maternal occupation.  **Covariates:**  Gender, age, ethnicity, maternal body mass index (BMI), maternal height, urban/rural residence.  **Potential mediators:**  Breastfeeding, maternal alcohol and smoking in pregnancy. | **Height:**  Stunting as defined by the 2006 WHO growth standard reference.  **Other(s):**  Overweight and wasting based on weight for length z scores (>2SD and <-2SD respectively). | Adjusted multivariate regression model for stunting showed statistically significant association with lower maternal education (β=-0.09, OR=0.92 (95% CI 0.89=0.94)), lower birth weight (β=-0.001, OR=0.99, 95% CI 0.99-0.99)), and female sex (β=0.047, OR=0.43 (95% CI 0.49-0.78)). No statistically significant associations were seen between stunting and maternal BMI, breastfeeding, wealth index, urban/rural residence and ethnicity.  Chi-squared tests did not show no significant associations between stunting and maternal alcohol and smoking during pregnancy. |
| **Bann et al. 2018**  Socioeconomic inequalities in childhood and adolescent body-mass index, weight, and height from 1953 to 2015: an analysis of four longitudinal, observational, British birth cohort studies.  England, Scotland, Wales. | **Design:**  Secondary analysis of four longitudinal observation birth cohort studies.  **Participants:**  Single births from British birth cohort studies from 1946, 1958, 1970 and 2001.  **Sample size:**  57,258 | **SES:**  Socioeconomic position as indicated by their father’s occupation.  Maternal education.  **Covariates:**  Ethnicity. | **Height:**  Height measurements at ages 7, 11 and 15 years.  **Other(s):**  Weight and BMI measurements at ages 7, 11 and 15 years. | Lower socioeconomic position was associated with shorter height at age 7, 11 and 15 years in all cohorts. This difference reduced in the later born cohorts.  At age 11 years the socioeconomic position slope index of inequality was -4.1 (95% CI -5.1 to -3.2) in 1946, -3.5 (95% CI -3.9 to -3.0) in 1958, -2.7 (95% CI -3.1 to -2.3) in 1970 and -1.2 (95% CI -1.7 to -0.6) in 2001. |
| **Batis et al. 2020**  Malnutrition prevalence among children and women of reproductive age in Mexico by wealth, education level, urban/rural area and indigenous ethnicity.  Mexico | **Design:**  Cross-sectional study.  **Participants:**  Children aged <5 years, females aged 11-49 years.  2012 Health and Nutritional Survey.  **Sample size:**  33,244 (total).  10,658 children <5 years.  22,586 (females aged 11-49 years). | **SES:**  Maternal education,  household wealth index.  **Covariates:**  Ethnicity, urban/rural residence. | **Height:**  Stunting as defined by 2006 WHO growth standards reference.  **Other(s):**  Overweight, obesity, wasting, underweight, anaemia (Hb<110g/l). | In a model with multivariable adjustment for confounding factors  there were statistically significant lower prevalence ratios (PR) of stunting in children under 5 years in children in high versus low wealth tertiles (PR 0.36, 95% CI 0.29-0.45), medium versus low maternal education (PR 0.55, 95% CI 0.47-0.64), urban versus rural residence (PR 0.83, 95% CI 0.72-0.97), and non-indigenous vs indigenous ethnicity (PR 0.54, 95% CI 0.45-0.64). |
| **Cardenas et al. 2022**  Mind the Gap! Socioeconomic Determinants of the Stunting Urban-Rural Gap for Children in Colombia.  Colombia | **Design:**  Cross sectional study.  **Participants:**  Children <5 years.  2015 National Nutritional Situation Survey.  **Sample size:**  8,557 | **SES:**  Maternal education, household wealth index.  **Covariates:**  Urban/rural residence, maternal BMI, sex, age, birth interval, maternal age.  **Potential mediators:**  Breastfeeding status, place of delivery, attendance at antenatal check-ups. | **Height:**  Stunting based on height-for-age Z score from WHO 2006 growth standards. | Rates of stunting were significantly higher in rural than urban areas (7.26% 95% CI 5.7%-8.8%). In a decomposition analysis 95.7% (95% CI 79.9%-111.6%) of this difference was explained by different in socioeconomic characteristics.  In an adjusted logistic regression analysis higher maternal education (no versus secondary education, β=-0.071, p<0.01), higher household wealth index (very poor vs very rich, β=-0.075, p<0.01), institutional delivery (β=-0.068, p<0.05), and attendance at four antenatal checks (β=-0.068, p<0.05) were all associated with lower rates of stunting. No statistically significant associations were seen between stunting and sex, maternal age, maternal BMI and birth interval. |
| **Flores et al. 2021**  Dietary patterns in Mexican preschool children are associated with stunting and overweight.  Mexico | **Design:**  Cross-sectional study.  **Participants:**  Children <5 years.  Mexican National Health and Nutritional Survey 2012.  **Sample size:**  1,112 | **SES:**  Household lead educational level, household wealth index, beneficiary of social welfare.  **Covariates:**  Gender, age, ethnicity, rural/urban residence, region.  **Potential mediators:**  Dietary information collected through a validated semi-quantitative-food-frequency questionnaire. | **Height:**  Stunting according to height-for-age WHO growth reference charts.  **Other(s):**  Overweight, obesity. | In a Poisson regression model after adjustment for sociodemographic factors stunting prevalence ratios were 2.04 times higher (95% CI 1.17-3.56) in children with highest tertile diets of fruit and vegetables consumption, 0.48 (95% CI 0.27-0.85) lower in children with the highest tertile of western diets, and 1.74 (95%CI 1.01-3.00) higher with the highest tertile of traditional diets. |
| **Hancock et al. 2016**  Socioeconomic variation in height: analysis of National Child Measurement Programme data for England.  England | **Design:**  Cross-sectional study.  **Participants:**  Children aged 4-5 years and 10-11 years attending state primary schools.  NCMP from 2008/2009 to 2012/2013.  **Sample size:**  1,213,230 | **SES:**  Income Deprivation Childhood Index (IDACI).  **Covariates:**  Ethnicity. | **Height:**  Mean height (cm) standard deviation score in each group measured at 4-5 and 10-11 years. | Mean height in White British children decreased by 0.2 Standard Deviation Score (SDS) between the least and most deprived quintiles. The difference in mean height in Asian children between the least and most deprived quintiles varied by 0.08 to 0.18 SDS. There was no statistically significant variation in height by deprivation in Black children. |
| **Karaoğlan & Saraçoğlu 2017**  Socio-Economic Factors Affecting Early Childhood Health: The Case of Turkey.  Turkey | **Design:**  Cross-sectional study.  **Participants:**  Children aged between 7-59 months.  Demographic Health Survey 2013.  **Sample size:**  1,811 | **SES:**  Maternal education, household wealth index.  **Covariates:**  Maternal height, ethnicity, birth order, region.  **Potential mediators:**  Nutrition index score, pre-school attendance, birth weight, breastfeeding status. | **Height:**  Stunting according to height-for-age z scores. | Multivariate regression analysis showed statistically significant correlation between greater birth weight (0.00138, p<0.01), urban living (0.648, p<0.05) and greater childhood height after adjustment for confounders. No association with childhood height and breastfeeding, wealth index, household size, maternal height, maternal education, pre-school attendance and nutrition index score. |
| **Leroy et al 2014.**  Maternal Education Mitigates the Negative Effects of Higher Income on the Double Burden of Child Stunting and Maternal Overweight in Rural Mexico.  Mexico | **Design:**  Cross-sectional study.  **Participants:**  Child (<5 years) mother (18-49 years) pairs from 235 rural communities in 2003/2004.  **Samples size:**  1,547 child mother pairs. | **SES:**  Household wealth index,  maternal education.  **Covariates:**  Maternal age, maternal height, maternal BMI, child sex, child age. | **Height:**  Height-for-age z scores.  **Other(s):**  Maternal overweight according to BMI, overweight mother stunted child pairs. | Linear regression analysis showed statistically significant associations between child height-for-age z score and maternal height (β=4.431, p<0.001) and combined maternal education and household wealth index (β=0.255, p=0.017) after adjustment for confounders. There was no association between height-for-age z scores and maternal education, and household wealth index after adjustment. |
| **Lopez-Arana et al. 2016**  Conditional cash transfers and the double burden of malnutrition among children in Colombia: a quasi-experimental study.  Colombia | **Design:**  Quasi-experimental study using a differences in differences approach.  **Participants:**  Children under 7 years in participating municipalities.  2^nd^ follow-up survey 2006/2006.  **Sample size:**  2,874 | **Intervention:**  Receiving a conditional cash transfer of 40,000 Colombian Pesos children attending vaccination, and growth and development check-ups (n=1,290).  **Controls:**  Children from matched control municipalities (n=1584).  **Covariates:**  Age, sex, maternal education, maternal age, maternal BMI, household income, region. | **Height:**  Stunting according to height-for-age z scores.  **Other(s):**  Thinness, overweight, obesity. | Baseline prevalence of stunting was 30·3%, in treatment municipalities and 27·9% in control municipalities.  The prevalence of stunting decreased over time but the intervention did not have a significant effect on these outcomes. |
| **Novotny et al. 2017**  Dual burden of malnutrition in US Affiliated Pacific jurisdictions in the Children’s Healthy Living Program.  USA | **Design:**  Cross-sectional study.  **Participants:**  Children aged 2-8 years from 51 communities in 11 USA affiliated Pacific Jurisdictions (Alaska, American Samoa, Commonwealth of the Northern Mariana Islands, Guam, Hawaii, Federated States of Micronesia, Marshall Islands, and Palau).  **Sample size:**  5,558 | **SES:**  Jurisdiction income.  **Covariates:**  Age, sex, ethnicity. | **Height:**  Stunting at birth according to length for age z scores and current stunting according to height for age z scores.  **Other:**  Obesity. | Those who were stunted at birth were less likely to be currently stunted than those who were not stunted at birth (OR = 0.19, 95% CI: 0.05–0.69).  Prevalence of stunting at birth and childhood stunting both varied significantly according to ethnicity (p<0.001).  Childhood stunting decreased significantly with increasing income by jurisdiction (low middle income jurisdictions 2.9% vs high income jurisdictions 1.0%, 𝒙^2^ p<0.05). |
| **Nunez & Perez 2021**  The Escape from Malnutrition of Chilean Boys and Girls: Height-for-Age Z Scores in Late XIX and XX Centuries.  Chile | **Design:**  Meta-analysis of observational studies.  **Participants:**  Chilean boys and girls aged 5-18 years born between 1877 and 2001 using data from 53 studies.  **Sample size:**  1,258 | **SES:**  Available for five of the studies in the meta-analysis – included SES based on area, school attended, and household education and occupation.  **Covariates:**  Time period. | **Height:**  Stunting as measured by height-for-age z scores below 2 standard deviations. | Significantly lower height-for-age in lower compared with higher SES groups across all measured time periods (p<0.01)(1900, 1948, 1991).  Equalities in height-for-age z scores according to SES converged over time from a difference of 1.50 in boy and 1.48 in girls in 1948, to 0.69 in boys and 0.68 in girls in 1991. |
| **Orr et al. 2021**  Regional differences in short stature in England between 2006 and 2019: A cross- sectional analysis from the National Child Measurement Programme.  England | **Design:**  Cross-sectional study.  **Participants:**  Children aged 4-5 years.  NCMP from 2006/2007 to 2018/2019.  **Samples size:**  7,062,071 | **SES:**  Index of multiple deprivation (IMD).  **Covariates:**  Sex, local authority area, and ethnicity. | **Height:**  Short stature according to height-for-age z scores according to UK WHO reference values. | In an adjusted logistic regression model short stature was linearly associated with greater deprivation according to IMD. Prevalence of stunting in the most deprived decile was double the rates in the least deprived decile (2.56% (95% CI 2.53–2.59) vs. 1.38% (95% CI 1.35–1.41)).  Ethnic heterogeneity in rates of stunting with the highest rates of stunting in Indian children (2.52% (95% CI 2.45–2.60)) and the lowest rates in black children (0.64% (95% CI 0.61–0.67).  Short stature declined in all areas from 2006-2010 to 2016-2019 (2.03% (95% CI 2.01–2.05) to 1.82% (95% CI 1.80–1.84). Declines in stunting in all IMD deciles. Largest declines in stunting in the most deprived deciles, however disparities in stunting according to deprivation remained. |
| **Osorio et al. 2018**  Socioeconomic context of the community and chronic child malnutrition in Colombia.  Colombia | **Design:**  Cross-sectional survey.  **Participants:**  Children <5 years from 3,528 communities.  National Demographic and Health Survey 2010.  **Sample size:**  11,448 | **SES:**  Maternal education, household wealth.  **Covariates:**  Sex, age, birth interval, maternal BMI, urban/rural residence.  **Potential mediators:**  Breastfeeding duration, access to health system. | **Height:**  Stunting, based on height-for-age z scores according to WHO 2006 standards. | Results from a multilevel logistic regression analysis with random effects modelling showed that stunting increased with age and male sex (OR 1.228, 95% CI 1.10-1.38, p<0.001), never breastfeeding (OR 1.488, 95% CI 1.01-2.18, p<0.05), birth interval <24 months (OR 1.283, 95% CI 1.05-1.57, p<0.05) maternal education (secondary vs none, OR 0.553, 95% CI 0.40-0.76, p<0.001), access to health system (low vs high, OR 1.385, 95% CI 1.19-1.62, p<0.001), and household wealth (Q1 very poor vs very rich Q5, OR 2.217, 95% CI 1.64-3.00) after adjustment for confounders. There was no association after adjustment between stunting and maternal BMI. |
| **Riedl et al. 2019**  Body mass index and height in 11- to 16-year-old Austrian students attending two different school types with divergent socioeconomic backgrounds.  Austria | **Design:**  Cross-sectional study.  **Participants:**  Students aged 11-16 years attending vocational directed secondary schools (VSS) and secondary academic schools (AHS).  **Sample size:**  4579 | **SES:**  Attendance at a low socioeconomic status VSS vs a high socioeconomic status AHS.  **Covariates:**  Regions, urban/rural residence. | **Height:**  Short stature according to height-for-age z scores.  **Other(s):**  Overweight/obesity according to BMI z scores. | The mean difference in height according to school type was 0.93cm in pupils at AHS compared to VSS after adjusting for region and urbanicity (p<0.001).  Short stature was more common in males and females attending VHS than AHS (4.0% and 3.0%, vs 1.3% and 1.9%). |
| **Sansón-Rosas et al. 2021**  Food insecurity and the double burden of malnutrition in Colombian rural household.  Colombia | **Design:**  Cross-sectional study.  **Participants:**  Children (<5 years) mother pairs.  Colombian National Nutritional Survey from 2015.  **Sample size:**  2350 | **SES:**  Maternal education, household wealth index.  **Covariates:**  Ethnicity, region, maternal age, birth interval, maternal height, number of children <5 years in household.  **Potential mediator:**  Pre-term birth,  household food security status using the Latin-American and Caribbean Food Security Scale. | **Height:**  Stunting according to child height-for-age z scores.  **Other(s):**  Maternal weight category according to BMI. | In an adjusted logistic regression stunting was associated with household size (≥5 vs ≤4, OR 1.81, 95% CI 1.29-2.53), number of children under 5 years in the household (2 vs 1, OR 3.61, 95% CI 2.40-5.45), pre-term birth (OR 1.64, 95% CI 1.15-2.35), and male sex (OR 1.45, 95% CI 1.11-1.88). There was no significant association with wealth index, maternal education, and food insecurity.  Food insecurity was significantly association with lower household wealth index and maternal education (p<0.001). |
| **Santa-Ramírez et al. 2023**  Small area vulnerability, household food insecurity and child malnutrition in Medellin, Colombia: results from a repeated cross-sectional study.  Colombia | **Design:**  Cross-sectional study.  **Participants:**  Children <5 years.  Living Standards Measurement Survey from 2017 and 2018.  **Sample size:**  7962 | **SES:**  Social vulnerability index.  **Covariates:**  Sex, age.  **Potential mediators:**  Household food insecurity as measured by an adaptation of the Food Insecurity Experience Scale (FIES). | **Height:**  Stunting based on height-for-age z scores according to 2006 WHO reference growth classifications.  **Other(s):**  Wasting, overweight. | In a mixed effects Poisson regression model deprivation was significantly associated with food insecurity with those in the highest quintile of deprivation more likely to experience food insecurity than the least deprived quintile (PR 1.9, 95% CI 1.42–2.57).  The prevalence ratio for stunting was 1.93 (95% CI 1.55–2.39) times higher in the least deprived quintile than the most deprived quintile. |
| **Santas & Eryurt 2020**  Distribution of child health indicators according to internal migration and various social variables in Turkey.  Turkey | **Design:**  Cross-sectional study.  **Participants:**  Children <5 years. Demographic and Health Surveys (1993, 1998, 2003, 2008, 2013).  **Sample size:**  40,321 | **SES:**  Maternal education.  **Covariates:**  Rural/urban, migration status, residence, region, sex, age.  **Potential mediator(s):**  Antenatal care. | **Height:**  Stunting according to height-for-age z score.  **Other(s):**  Birthweight, overweight, obesity. | In the adjusted logistic regression analysis stunting was significantly higher in rural areas compared to urban areas (OR 1.835, p<0.01), in mothers with lower maternal education, incomplete primary education (OR 3.249, p<0.01) and primary education (OR 1.665, p<0.01) compared to secondary education and higher.  In an adjusted logistic regression for low birth weight there was significant associations with lower maternal education (incomplete primary education vs complete secondary education, OR 1.922, p<0.01), rural residence (OR 1.152, p<0.01), and no antenatal care (OR 1.339, p<0.01). |
| **Shackleton et al. 2015**  Trends and socioeconomic disparities in preadolescent’s health in the UK: evidence from two birth cohorts 32 years apart.  United Kingdom | **Design:**  Comparative cohort study.  **Participants:**  Using data from sweep 2 (1980) of the British Cohort study (1970) and sweep 5 (2012) of the Millennium Cohort Study (2000/2001).  **Sample size:**  13,554 (1980)  13,497 (2012) | **SES:**  Household income. | **Height:**  Stunting measured by height-for-age z scores.  **Other(s):**  General health, underweight, overweight, obesity allergy, childhood infectious diseases, health service use, smoking status. | Mean proportion of stunting decreased significantly between the two birth cohorts (-0.18, 95% CI -0.19 to -0.17).  Inequalities in stunting decreased significantly from between the two birth cohorts from β=-0.29 (95% CI -0.33 to -0.24) in the in 1980 to β=-(0.09, 95% CI -0.14, to -0.04) in 2012 (𝒙^2^=16.85, p<0.001). |
| **Shamah-Levy et al. 2017**  Food insecurity and maternal–child nutritional status in Mexico: cross- sectional analysis of the National Health and Nutrition Survey 2012.  Mexico | **Design:**  Cross-sectional study.  **Participants:**  Preschool children (<5 years) and school children (5-11 years).  Mexican National Health and Nutrition Survey 2012.  **Sample size:**  12,268 | **SES:**  Maternal education,  Socioeconomic status index which is a composite of a variety of measures.  **Covariates:**  Sex, urban/rural residence, region, benefits from food assistance programme.  **Potential mediators:**  Household food insecurity as measured by the Latin American and Caribbean Food security Scale. | **Height:**  Stunting according measured by height-for-age z scores according to WHO growth charts.  **Other (s):**  Overweight, obesity. | Severe household food insecurity was significantly more prevalent in the lowest (18.0% (95% CI 15.1- 21.4)) than the highest socioeconomic quintiles (2.8% (95% CI 1.8-4.6%)).  In the adjusted logistic regression model moderate (OR 1.66, 95% CI 1.0-2.7, p=0.036) and severe (OR 1.99, 95% CI 1.2-3.3, p=0.007) household food insecurity, rural residence (OR 1.42, 95% CI 1.1-1.8) and lower maternal education (primary vs secondary, OR 0.34, 95% CI 0.2-0.6, p<0.001) were significantly associated with stunting in children under 5 years. |
| **Silverwood et al. 2016**  Pathways between Socioeconomic Disadvantage and Childhood Growth in the Scottish Longitudinal Study, 1991–2001.  Scotland | **Design:**  Cohort study.  **Participants:**  A random sample of children born between 1991 and 2001 in Scotland.  **Sample size:**  16,628 | **SES:**  Scottish Index or Multiple Deprivation (SIMD), synthetic weekly income, maternal education.  **Covariates:**  Sex, year of birth, ethnicity, maternal age, maternal parity.  **Potential mediator:**  Birth weight. | **Height:** Length/height-for-age at 6-8 weeks, 8-9 weeks, 21-24 months, 39-42 months and 48 months. | An estimated regression coefficients model showed a significantly lower height in children at 48 months with lower maternal education (no qualifications vs degree, β=-0.98, 95% CI -1.22 to -0.75, p<0.001), lower SIMD quartile (Q1 vs Q4, β=-0.91, 95% CI -1.10 to -0.72, p<0.001) and lower synthetic income quartile (Q1 vs Q4, β=-0.75, 95% CI -0.97 to -0.54, p<0.001).  In a traditional mediation analysis, the effects of maternal education, SIMD and synthetic income were mediated when adjusted for low birth weight by 43%, 40% and 88% respectively.  In a counterfactual based mediation analysis, the effects of maternal education, SIMD and synthetic income on childhood height were mediated by 20%, 20% and 16% respectively when adjusted for low birth weight. |
