# Supplementary material for: Exploration of factors mediating the effect of socioeconomic deprivation on rates of childhood stunting in Sefton: an ecological analysis
Source: J Public Health (Oxf). 2026 Jul 6;48(3):788–96. doi: 10.1093/pubmed/fdag047 (PMC13531711; doi:10.1093/pubmed/fdag047)
Supplement: Supplementary file 1 [file supplementary_material_fdag047.zip › Appendix 2 Data sources for variables used in analyses.docx]

# Appendix 2: Data sources for variables used in analysis

Table 5: Data sources for variables identified by literature reviews and used in analysis

| Variable type | Variable | Indicator | Level of data | Year | Data source |
| --- | --- | --- | --- | --- | --- |
| Outcome | Childhood stunting | Height-for-age z score greater than two standard deviations below the population median as defined by the WHO growth reference standard, based on heights measured at reception (4-5 years) and year 6 (10-11 years). | Individual data from NCMP. | 2013/2014 to 2022/2023 | NCMP dataset for Sefton metropolitan area provided by Sefton Council. |
| Exposure | Socioeconomic deprivation | IDACI score separated into Sefton quintiles. | 2011 LSOA. | 2019 | Publicly available data from the Ministry of Housing, Communities and Local Government (2018 to 2021). |
| Mediators | Low birth weight | Proportion of babies born with weight <2500 gram by LSOAs within Sefton across the time period July 2023 to July 2024. | 2011 MSOA. | 2021/2022 to 2023/2024 | Data provided from NHS Cheshire and Merseyside Integrated Care Board. |
|  | Preterm birth | Proportion of babies born with before 37 weeks gestation by LSOAs within Sefton across the time period July 2023 to July 2024. | 2011 MSOA. | 2021/2022 to 2023/2024 | Data provided from NHS Cheshire and Merseyside Integrated Care Board. |
|  | Access to healthcare | Healthcare domain score component of the Index of Access to Healthy Hazards and Assets (AHAH) version 2 | 2011 LSOA. | 2017 | Publicly available from the Consumer Data Research Centre. |
|  | Food insecurity | Priority Place for Food Index version 2 total score by LSOA. | 2021 LSOA. | 2024 | Publicly available index produced in collaboration between the University of Leeds and Which?. |
|  | Breastfeeding | Percentage breastfeeding (exclusive and mixed) at 6 to 8 weeks after birth by electoral ward. | Electoral ward. | 2022/2023 | Data provided from NHS Cheshire and Merseyside Integrated Care Board. |
| Confounders | Ethnicity | Percentage of the population from six ethnic group categories (Asian, Black, Mixed, White British, White Other, and Other) by each LSOA. | 2011 LSOA | 2021 | Data publicly available from the Office for National Statistics. |
|  | Rural/urban residence | Rural or rural LSOA as defined by the Office for National Statistics. | Individual-level data from NCMP. | 2013/2014 to 2022/2023 | NCMP dataset for Sefton metropolitan area provided by Sefton Council. |
|  | Household size | Percentage of household with overcrowding based on room occupancy. | 2011 MSOA. | 2011 | Publicly available from the Office for Health Improvement and Disparities. |
