# Supplementary material for: Exploration of factors mediating the effect of socioeconomic deprivation on rates of childhood stunting in Sefton: an ecological analysis
Source: J Public Health (Oxf). 2026 Jul 6;48(3):788–96. doi: 10.1093/pubmed/fdag047 (PMC13531711; doi:10.1093/pubmed/fdag047)
Supplement: Supplementary file 1 [file supplementary_material_fdag047.zip › Appendix 3 Changes in rates of stunting over time.docx]

# Appendix 3: Changes in rates of stunting over time

Percenatge of pupils with stunted growth from 2013/2014 to 2022/2023

Percentage with stunted growth

|  | Reception | Year 6 |
| --- | --- | --- |
| 2013/2014 | 1.7673 | 1.72348 |
| 2014/2015 | 1.61349 | 0.917782 |
| 2015/2016 | 1.54426 | 0.892193 |
| 2016/2017 | 1.48423 | 1.25323 |
| 2017/2018 | 1.73368 | 0.962963 |
| 2018/2019 | 1.85747 | 1.39735 |
| 2019/2020 | 1.044 | 1.47194 |
| 2020/2021 | 1.4218 | 0.888889 |
| 2021/2022 | 1.71625 | 0.692521 |
| 2022/2023 | 2 | 0.758671 |

*Figure 5: Percentage of pupils with stunted growth from 2013/2014 to 2022/2023*
