# Supplementary material for: Exploration of factors mediating the effect of socioeconomic deprivation on rates of childhood stunting in Sefton: an ecological analysis
Source: J Public Health (Oxf). 2026 Jul 6;48(3):788–96. doi: 10.1093/pubmed/fdag047 (PMC13531711; doi:10.1093/pubmed/fdag047)
Supplement: Supplementary file 1 [file supplementary_material_fdag047.zip › Appendix 4 The relationship between IDACI quintiles and childhood stunting.docx]

# Appendix 4: The correlation between IDACI quintiles and childhood stunting in reception and year 6 pupils

Percentage of pupils with stunting by IDACI quintile

Percenatge with stunting (%)

| IDACI quintile | Reception | Year 6 |
| --- | --- | --- |
| 1 | 2.45713 | 1.62647 |
| 2 | 1.65306 | 1.14715 |
| 3 | 1.37398 | 0.772986 |
| 4 | 1.18597 | 1.07456 |
| 5 | 1.38236 | 0.815091 |

*Figure 6: Percentage of pupils with stunted growth according to IDACI quintiles in Sefton*

Table 6: Logistic regression model for the association between IDACI quintile and childhood stunting adjusted for confounders in reception pupils

|  | OR | OR Upper 95% CI | OR Lower 95% CI | p-value |
| --- | --- | --- | --- | --- |
| **IDACI Quintiles** | | | | |
| 1 (most deprived) | 1.60 | 1.10 | 2.35 | **0.016** |
| 2 | 1.18 | 0.82 | 1.72 | 0.380 |
| 3 | 0.95 | 0.65 | 1.40 | 0.811 |
| 4 | 0.83 | 0.55 | 1.24 | 0.369 |
| 5 (reference) | 1.00 |  |  |  |
| **Confounders** | | | | |
| **Ethnicity** | | | | |
| White British (Reference) | 1.00 |  |  |  |
| White Other | 0.97 | 0.93 | 1.01 | 0.186 |
| Asian | 0.91 | 0.81 | 1.01 | 0.085 |
| Black | 1.02 | 0.86 | 1.19 | 0.808 |
| Mixed | 0.97 | 0.82 | 1.15 | 0.739 |
| Other | 1.05 | 0.89 | 1.22 | 0.567 |
| **Housing** | | | | |
| Urban/rural residence | 1.72 | 0.54 | 10.4 | 0.449 |
| Overcrowded housing | 1.05 | 1.01 | 1.09 | **0.023** |

Table 7: Logistic regression model for the association between IDACI quintile and childhood stunting adjusted for confounders in year 6 pupils

|  | OR | OR Upper 95% CI | OR Lower 95% CI | p-value |
| --- | --- | --- | --- | --- |
| **IDACI Quintiles** | | | | |
| 1 (most deprived) | 2.02 | 1.28 | 3.25 | **0.003** |
| 2 | 1.37 | 0.88 | 2.16 | 0.165 |
| 3 | 0.96 | 0.60 | 1.53 | 0.852 |
| 4 | 1.29 | 0.83 | 2.01 | 0.256 |
| 5 (reference) | 1.00 |  |  |  |
| **Confounders** | | | | |
| **Ethnicity** | | | | |
| White British (Reference) | 1.00 |  |  |  |
| White Other | 1.04 | 0.99 | 1.10 | 0.119 |
| Asian | 0.94 | 0.83 | 1.06 | 0.359 |
| Black | 0.76 | 0.57 | 0.99 | 0.053 |
| Mixed | 0.96 | 0.77 | 1.18 | 0.676 |
| Other | 1.13 | 0.91 | 1.37 | 0.247 |
| **Housing** | | | | |
| Urban/rural residence | 1.10 | 0.34 | 6.69 | 0.898 |
| Overcrowded housing | 0.99 | 0.94 | 1.05 | 0.847 |
