# Supplementary material for: Exploration of factors mediating the effect of socioeconomic deprivation on rates of childhood stunting in Sefton: an ecological analysis
Source: J Public Health (Oxf). 2026 Jul 6;48(3):788–96. doi: 10.1093/pubmed/fdag047 (PMC13531711; doi:10.1093/pubmed/fdag047)
Supplement: Supplementary file 1 [file supplementary_material_fdag047.zip › Appendix 5 Mediation analysis.docx]

# Appendix 5: Exploratory mediation-style analysis

Table 8: Adjusted logistic regression models examining the effect of hypothesised mediatiors on the relationship between IDACI quintiles and childhood stunting in reception pupils

|  | Model 1: Adjusted for confounders | | | | Model 2: Adjusted for confounders and hypothesised mediators | | | |
| --- | --- | --- | --- | --- | --- | --- | --- | --- |
|  | OR | OR Upper 95% CI | OR Lower 95% CI | p-value | OR | OR Upper 95% CI | OR Lower 95% CI | p-value |
| **IDACI Quintiles** | | | | | | | | |
| 1 (most deprived) | 1.44 | 0.97 | 2.18 | 0.0749 | 1.57 | 0.86 | 2.87 | 0.143 |
| 2 | 1.02 | 0.68 | 1.55 | 0.916 | 1.12 | 0.67 | 1.91 | 0.652 |
| 3 | 0.82 | 0.54 | 1.26 | 0.364 | 0.88 | 0.54 | 1.46 | 0.627 |
| 4 | 0.75 | 0.48 | 1.16 | 0.196 | 0.76 | 0.47 | 1.22 | 0.257 |
| 5 (reference) | 1.00 |  |  |  | 1.00 |  |  |  |
| **Confounders** | | | | | | | | |
| **Ethnicity** | | | | | | | | |
| White British (Reference) | 1.00 |  |  |  | 1.00 |  |  |  |
| White Other | 0.98 | 0.93 | 1.03 | 0.387 | 0.97 | 0.92 | 1.02 | 0.179 |
| Asian | 0.89 | 0.79 | 1.00 | **0.0485** | 0.87 | 0.77 | 0.98 | **0.0211** |
| Black | 1.00 | 0.84 | 1.18 | 0.987 | 1.03 | 0.85 | 1.22 | 0.745 |
| Mixed | 0.99 | 0.83 | 1.17 | 0.876 | 0.95 | 0.79 | 1.14 | 0.583 |
| Other | 1.05 | 0.89 | 1.22 | 0.548 | 1.02 | 0.86 | 1.20 | 0.794 |
| **Housing** | | | | | | | | |
| Urban/rural residence | 0.68 | 0.45 | 12.2 | 0.710 | 0.35 | 0.064 | 6.57 | 0.327 |
| Overcrowded housing | 1.04 | 0.99 | 1.08 | 0.106 | 1.03 | 0.98 | 1.08 | 0.184 |
| **Hypothesised mediators** | | | | | | | | |
| **Birth complication** | | | | | | | | |
| Low birth weight | **-** | - | - | - | 1.05 | 0.98 | 1.11 | 0.16 |
| Preterm birth | **-** | - | - | - | 0.97 | 0.90 | 1.04 | 0.389 |
| **Healthcare** | | | | | | | | |
| Health domain AHAHv2 mean score | - | - | - | - | 0.98 | 0.96 | 1.00 | 0.0704 |
| **Food insecurity** | | | | | | | | |
| PPFIv2 mean score | - | - | - | - | 1.00 | 1.00 | 1.00 | 0.882 |
| **Breastfeeding** | | | | | | | | |
| Breastfeeding at 6-8 weeks | **-** | **-** | - | - | 1.00 | 0.99 | 1.02 | 0.532 |

p-values <0.05 shown in bold

Table 9: Adjusted logistic regression model examining the effect of mediation on the relationship between IDACI quintiles and childhood stunting in year 6 pupils

|  | Model 1: Adjusted for confounders | | | | Model 2: Adjusted for confounders and hypothesised mediators | | | |
| --- | --- | --- | --- | --- | --- | --- | --- | --- |
|  | OR | OR Upper 95% CI | OR Lower 95% CI | p-value | OR | OR Upper 95% CI | OR Lower 95% CI | p-value |
| **IDACI Quintiles** | | | | | | | | |
| 1 (most deprived) | 1.81 | 1.10 | 3.09 | **0.0233** | 1.20 | 0.56 | 2.60 | 0.640 |
| 2 | 1.23 | 0.74 | 2.10 | 0.424 | 0.97 | 0.51 | 1.89 | 0.939 |
| 3 | 0.78 | 0.44 | 1.38 | 0.388 | 0.68 | 0.35 | 1.32 | 0.254 |
| 4 | 1.28 | 0.78 | 2.16 | 0.342 | 1.21 | 0.70 | 2.14 | 0.893 |
| 5 (reference) | 1.00 |  |  |  | 1.00 |  |  |  |
| **Confounders** | | | | | | | | |
| **Ethnicity** | | | | | | | | |
| White British (Reference) | 1.00 |  |  |  | 1.00 |  |  |  |
| White Other | 1.04 | 0.98 | 1.10 | 0.176 | 1.05 | 0.99 | 1.12 | 0.107 |
| Asian | 0.98 | 0.85 | 1.10 | 0.701 | 0.96 | 0.83 | 1.09 | 0.515 |
| Black | 0.82 | 0.62 | 1.05 | 0.147 | 0.80 | 0.59 | 1.04 | 0.115 |
| Mixed | 0.93 | 0.74 | 1.16 | 0.515 | 0.94 | 0.75 | 1.18 | 0.612 |
| Other | 1.04 | 0.88 | 1.34 | 0.410 | 1.05 | 0.87 | 1.34 | 0.433 |
| **Housing** | | | | | | | | |
| Urban/rural residence | 0.47 | 0.10 | 8.47 | 0.464 | 0.30 | 0.05 | 5.74 | 0.270 |
| Overcrowded housing | 1.00 | 0.94 | 1.05 | 0.902 | 0.98 | 0.92 | 1.03 | 0.459 |
| **Hypothesised mediators** | | | | | | | | |
| **Birth complication** | | | | | | | | |
| Low birth weight | **-** | - | - | - | 1.04 | 0.96 | 1.12 | 0.322 |
| Preterm birth | **-** | - | - | - | 1.01 | 0.92 | 1.10 | 0.893 |
| **Healthcare** | | | | | | | | |
| Access to health care (Health Domain AHAHv2 Score) | - | - | - | - | 0.99 | 0.96 | 1.01 | 0.412 |
| **Food insecurity** | | | | | | | | |
| Food insecurity (PPFIv2 Score) | - | - | - | - | 1.00 | 1.00 | 1.00 | 0.385 |
| **Breastfeeding** | | | | | | | | |
| Breastfeeding at 6-8 weeks | **-** | **-** | - | - | 0.99 | 0.97 | 1.01 | 0.263 |

Table 10: Proportion eliminated after adjustment for ecological indicators consistent with hypothesised pathways

|  | Total effects OR in model 1* | Direct effect OR in model 2** | Proportion eliminated |
| --- | --- | --- | --- |
| Reception | 1.44 (95% CI 0.97, 2.18) | 1.57 (95% CI 0.86, 2.87) | -29.5% |
| Year 6 | 1.81 (95% CI 1.10, 3.09) | 1.20 (95% CI 0.56, 2.60) | 75.3% |

*****Adjusted for confounders: ethnicity, rural/urban residence, household overcrowding, **Adjusted for confounders and hypothesised mediators: ethnicity, rural/urban residence, household overcrowding, food insecurity, healthcare access, preterm birth, low birth weight, breastfeeding
