# Supplementary material for: Exploration of factors mediating the effect of socioeconomic deprivation on rates of childhood stunting in Sefton: an ecological analysis
Source: J Public Health (Oxf). 2026 Jul 6;48(3):788–96. doi: 10.1093/pubmed/fdag047 (PMC13531711; doi:10.1093/pubmed/fdag047)
Supplement: Supplementary file 1 [file supplementary_material_fdag047.zip › Appendix 6 Differences in missing data.docx]

**Appendix 6: Differences in children excluded from exploratory mediation analysis**

Table 11: Differences between children included in the exploratory mediation-style analysis and those excluded due to missing data

|  | Included in mediation analysis models  (n=38490) | Not included in mediation analysis models (n= 7972) | p-value |
| --- | --- | --- | --- |
| **Stunting** | | | |
| Percentage with stunting (%) | 1.46% (n=561) | 1.02% (n=81) | **<0.0005*** |
| **IDACI Quintiles** | | | |
| 1 (most deprived) | 28.59% (n=11006) | 0.00% (n=0) | **<0.0005**** |
| 2 | 21.51% (n=8279) | 20.15% (n=1606) | **<0.0005**** |
| 3 | 18.93% (n=7286) | 23.2% (n=1851) | **<0.0005**** |
| 4 | 17.42% (n=6705) | 22.8% (n=1818) | **<0.0005**** |
| 5 | 13.55% (n=5214) | 33.8% (n=2697) | **<0.0005**** |
| **Ethnicity** | | | |
| White British (%) | 91.57% | 94.08% | **<0.0005***** |
| White Other (%) | 4.08% | 2.79% | **<0.0005***** |
| Asian (%) | 1.56% | 1.13% | **<0.0005***** |
| Black (%) | 0.59 | 0.26% | **<0.0005***** |
| Mixed (%) | 1.49% | 1.33% | **<0.0005***** |
| Other (%) | 0.71% | 0.40% | **<0.0005***** |
| **Housing** | | | |
| Rural residence (%) | 0.23% | 4.68% | **<0.0005*** |
| Urban residence (%) | 99.77% | 95.32% | **<0.0005*** |
| Overcrowded housing (%) | 5.00% | 2.33% | **<0.0005***** |
| **Breastfeeding** | | | |
| Breastfeeding at 6-8 weeks (%) | 36.4% | 45.4% | **<0.0005***** |
| **Food insecurity** | | | |
| PPFIv2 score (higher score indicates higher food insecurity) | 10901 | 16450 | **<0.0005***** |
| **Access to healthcare** | | | |
| Health domain score AHAHv2 (lower score indicates better access to healthcare) | 7.94 | 15.64 | **<0.0005***** |

*p-value calculated using Chi-squared test, p-value calculated using Fisher’s Exact test, ***p-value calculated using t-test, p-values <0.05 shown in bold
